# Major Histocompatibility Complex Immunogenetic Diversity Differs Substantially Across Sea Turtle Species and Genomic Regions

**DOI:** 10.1093/gbe/evag008

**Published:** 2026-01-23

**Authors:** Katherine R Martin, Jamie L Adkins, Vipheaviny Chea, Christine M Sarkis, Katrina F Phillips, Anna M Forsman, Erin E Seney, Lisa M Komoroske, Kate L Mansfield, Anna E Savage

**Affiliations:** Department of Biology, University of Central Florida, Orlando, FL, USA; Department of Environmental Conservation, University of Massachusetts, Amherst, MA, USA; Department of Environmental Conservation, University of Massachusetts, Amherst, MA, USA; Translational Immunogenomics Lab, Dana-Farber Cancer Institute, Boston, MA, USA; Department of Biology, University of Central Florida, Orlando, FL, USA; Department of Biology, University of Central Florida, Orlando, FL, USA; Department of Environmental Conservation, University of Massachusetts, Amherst, MA, USA; Department of Biology, University of Central Florida, Orlando, FL, USA; Department of Biology, Colby College, Waterville, ME, USA; Department of Biology, University of Central Florida, Orlando, FL, USA; Department of Environmental Conservation, University of Massachusetts, Amherst, MA, USA; Department of Biology, University of Central Florida, Orlando, FL, USA; Department of Biology, University of Central Florida, Orlando, FL, USA

**Keywords:** marine turtle, MHC, immunogenetics, Chelonioidea, pathogen-mediated balancing selection, comparative immunogenetics

## Abstract

Major histocompatibility complex (MHC) immune loci illustrate how natural selection shapes functional genetic diversity in wild populations. Balancing selection favors high MHC diversity within individuals and populations that persists beyond speciation, leading to shared allelic lineages among taxa. However, some vertebrates show markedly lower allelic diversity and even the loss of entire MHC gene classes. Variation in life history and disease prevalence makes sea turtles an important group for studying interspecific MHC diversity, but this has been minimally explored. We sequenced class I and class II MHC genes in over 300 individuals from loggerhead (*Caretta caretta*), green (*Chelonia mydas*), leatherback (*Dermochelys coriacea*), and Kemp's ridley (*Lepidochelys kempii)* sea turtles. We recovered 162 class I and 308 class II functionally distinct alleles, many of which were shared among species. Codon usage analyses suggest that the shared alleles have been maintained through speciation. High allele counts and evidence of diversifying and positive selection suggest balancing selection maintains considerable MHC diversity in sea turtles. However, we found two notable exceptions: (i) *D. coriacea* had extremely low nucleotide and allelic diversity across all MHC loci and (ii) one MHC class II gene copy on a different chromosome from the core MHC genomic region showed little evidence of positive selection and almost no genetic variability among all four species, suggesting an atypical and potentially functionally distinct gene. Our study finds notably high allelic and nucleotide diversity in sea turtle MHC promoted by balancing selection, yet evolutionary pressures vary considerably between species and gene copies.

Significance The major histocompatibility complex (MHC) genes are members of a highly variable gene family critical to pathogen recognition in the vertebrate acquired immune system. Despite their importance, we lack studies quantifying MHC variation in reptiles, including those simultaneously comparing MHC evolution between species and between gene copies. We find balancing selection has driven high MHC class I and MHC class II diversity in three sea turtle species and has contributed to the retention and maintenance of alleles that were likely present in the common ancestor of all extant sea turtles. However, leatherback sea turtles have much lower MHC class I and class II diversity, and all sea turtle species studied here possess an atypical, low diversity MHC class II gene copy. This study demonstrates that MHC evolution varies considerably and in unexpected ways between species and gene copies, highlighting the importance of measuring functional MHC diversity at intra- and interspecific levels in order to draw conclusions about adaptive immunity.

## Introduction

Immune responses against pathogens are critical components of organismal survival. The study of immune-associated loci offers insight into how evolutionary mechanisms, such as natural selection and genetic drift, interact to shape genetic diversity in wild populations (Piertney and Oliver 2006). Characterizing standing immunogenetic variation in species of conservation concern can also inform population management, especially for populations experiencing infectious diseases (Sommer 2005). The major histocompatibility complex (MHC) is the most polymorphic vertebrate gene family (Ohta 1983; Radwan et al. 2020) and is a model for how functionally important loci evolve under a suite of selective forces, including pathogen-mediated balancing selection (Spurgin and Richardson 2010; Radwan et al. 2020).

Classical MHC receptors bind proteins derived either from pathogens or from self peptides. Non-self peptides are presented to T lymphocytes, initiating an acquired immune response against foreign molecules (Murphy and Weaver 2017). MHC class I (MHCI) proteins bind intracellular antigens, such as those from viruses, whereas MHC class II (MHCII) proteins bind extracellular antigens, such as those from bacteria and fungi. Functional MHCI and MHCII proteins have peptide binding grooves where antigens are bound. In MHCI, the peptide binding groove is comprised of the α chain, encoded by the MHCIA genes. The MHCII peptide binding groove is composed of an α chain and a β chain, encoded by the MHCIIA genes and MHCIIB genes, respectively (Hughes and Yeager 1998; Murphy and Weaver 2017).

MHC loci are often highly variable in both number of gene copies and number of alleles. MHC genes evolve according to a birth–death process, where gene families expand via gene duplication and contract via pseudogenization or gene deletion (Nei and Rooney 2005). The result is variation in the number of MHC gene copies between even closely related species, referred to as copy number variation (CNV). For example, there is substantial CNV across the avian tree of life driven by either gene duplication and deletion, or by the expansion and contraction of the MHC region (Minias et al. 2019). Chickens (*Gallus gallus*) have a “minimal essential” MHC region with only one expressed gene at both MHCI and MHCII (Kaufman et al. 1999), whereas passerine birds such as the sedge warbler (*Acrocephalus schoenobaenus*) have up to 32 MHCI loci (Biedrzycka et al. 2017). In turn, each of these gene copies may be highly polymorphic. Extreme examples of this include the hundreds of MHCIA alleles present in a Polish population of sedge warblers as a result of pathogen-mediated balancing selection (Biedrzycka et al. 2018). In particular, amino acids comprising the peptide binding groove (eg MHCI exon 3 and MHCIIB exon 2) are frequently under strong positive selection across multiple taxa (eg Savage and Zamudio 2011; Jaratlerdsiri et al. 2014; Minias et al. 2021), highlighting their adaptive value in pathogen surveillance.

Balancing selection can also promote the retention of similar sequence motifs, similar alleles, and even identical alleles across species, genera (Lenz et al. 2013; Kaesler et al. 2017; Martin et al. 2022), and even families (Go et al. 2002). Three primary evolutionary scenarios may explain MHC allelic similarity: co-ancestry (i.e. ancestral polymorphism), convergent evolution, and adaptive introgression. In the co-ancestry scenario, balancing selection preserves ancestral MHC alleles in multiple lineages after speciation, likely because of the adaptive value of rare MHC variants in fighting disease (Spurgin and Richardson 2010; Těšický and Vinkler 2015). Under this scenario, allelic similarity that has evolved from common descent is referred to as trans-species polymorphism (Klein 1987). By contrast, convergent evolution can independently produce similar MHC alleles due to shared post-speciation selective pressures, resulting in MHC alleles with similar pathogen binding properties (Yeager and Hughes 1999). Finally, adaptive introgression may also explain MHC allelic similarity between species, again due to the fitness benefits of rare or novel MHC alleles introduced via hybridization among species (Gaczorek et al. 2023).

Sea turtles are among the major reptile clades with minimal information on MHC diversity and evolution, with prior work characterizing only MHCI loci across two species (Stiebens et al. 2013; Martin et al. 2022; Adkins et al. 2025). Variation in ecology, habitat use, and disease prevalence make sea turtles important for studying interspecific MHC diversity. For example, leatherback sea turtles (*Dermochelys coriacea*), the only extant member of Family *Dermochelydiae*, subsist on gelatinous prey, are almost entirely pelagic, are capable of facultative endothermy (Davenport et al. 1990), and do not undertake an ontogenetic niche shift (Bolten 2003). This is in stark contrast to some of the hard-shelled species in the *Cheloniidae* family that range from carnivorous to primarily herbivorous, and rely heavily on nearshore environments after an initial pelagic dispersal stage (Musick and Limpus 1997; Bolten 2003). Disease prevalence and severity also varies within *Cheloniidae*, with juvenile green sea turtles (*Chelonia mydas*) having high prevalence of the neoplastic tumor disease fibropapillomatosis compared to juvenile loggerhead sea turtles (*Caretta caretta*) even at the same sampling site (Martin et al. 2022). Characterizing the genetic underpinnings of sea turtle immune system function is relevant to understanding their ability to fight contemporary and future disease, especially in light of the multiple stressors they face, including novel disease emergence due to climate change and pollution (Fuentes et al. 2023). While initial studies indicate that *Ca. caretta* and *Ch. mydas* have high MHCI diversity with dozens of alleles (Stiebens et al. 2013; Martin et al. 2022; Adkins et al. 2025), we do not know whether this pattern is true of other species of sea turtles, and no population- or species-level studies of MHCII yet exist for any species of sea turtle.

In this study, we characterized diversity and evolutionary patterns of MHCI and MHCII loci across four species representative of extant sea turtle subfamilies and life histories: *Ca. caretta* (*Carettinae)*, *Lepidochelys kempii* (*Carettinae)*, *Ch. mydas* (*Cheloniinae*), and *D. coriacea* (*Dermochelyinae*). Our overarching goal was to quantify MHC diversity, understand its evolutionary drivers, and identify differences among species in this ancient reptile clade containing taxa with variable ecologies and disease pressures. We specifically focused on peptide-binding exons to understand how positive selection has shaped MHC evolution and function in sea turtles, as well as to compare MHC diversity across species. We identified the mode and tempo of MHC evolution in sea turtles by reconstructing gene trees and tested whether co-ancestry or convergent evolution contributes to allelic similarity between species. We measured positive selection within specific MHC gene lineages and at amino acid positions among recovered MHC alleles. We also described an MHC gene unlinked from the main MHC chromosomal region and tested for differences in evolutionary patterns at this locus. Together, these analyses revealed the shared and distinct evolutionary forces that have shaped immunogenetic diversity in four species of sea turtles, informing predictions of reptile immune gene evolution.

## Results

### MHC Allelic Diversity Differs Between Genomic Regions and Species

**Fig. 1.**
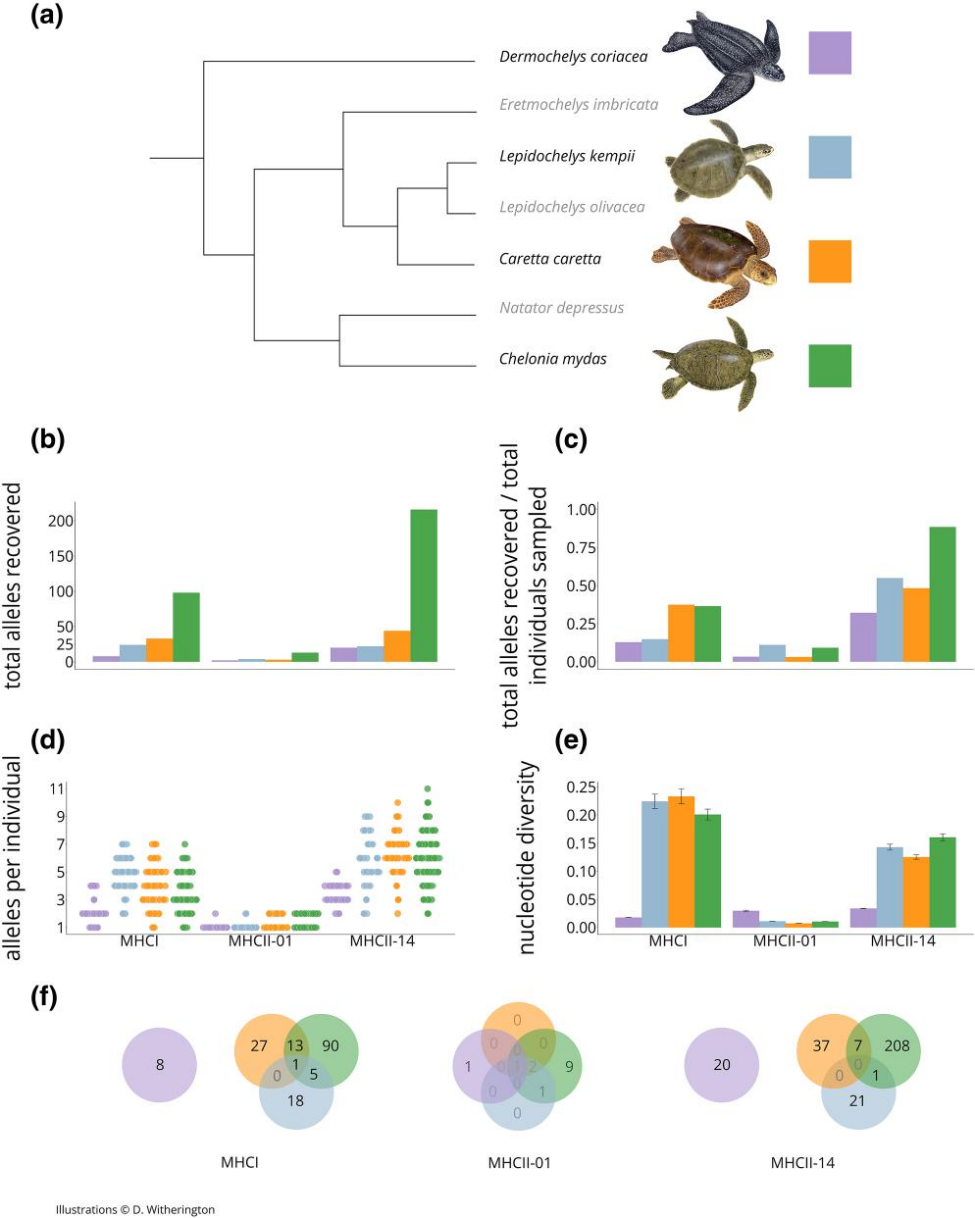
a) cladogram of extant sea turtle species, with sampled species denoted with illustration and colored box. Metrics of allelic diversity and count are presented by species (color) and by MHC region, demonstrating total alleles recovered (b); total alleles recovered per number of turtles sampled (c); the range of alleles recovered per individual (d); and nucleotide diversity of recovered alleles with variance (whiskers) (e). Venn diagrams representing shared alleles across species and MHC regions (includes MHCI alleles from Stiebens et al. 2013; Adkins et al. 2025) (f). Sea turtle illustrations copyright Dawn Witherington.

We genotyped three MHC regions from over 300 sea turtles (Fig. 1, Table 1): MHC class IA, MHC class IIB on the main MHC region on chromosome 14, and MHC class IIB on chromosome 1. We generally reached allelic saturation when accounting for unequal sample sizes across our four species (Fig. S4a–c). We had excellent repeatability across sequencing runs for both MHC class IIB regions we amplified, but only moderate repeatability for MHC class IA, suggesting allelic dropout and an underestimation of allele counts (Tables S2–S4). The MHC class IA exon 3 that we amplified (hereafter MHCI) was highly polymorphic with 162 total alleles (22 novel alleles; 1 to 7 alleles per individual). The MHC class IIB exon 2 region on chromosome 14 (MHCII-14) was even more polymorphic with 294 alleles (1 to 11 per individual). In contrast, we recovered only 14 alleles (1 to 2 per individual) from the MHC class IIB exon 2 region on chromosome 1 (MHCII-01) (Fig. 1c). MHCII-01 also had far lower nucleotide diversity compared to MHCI or MHCII-14 (Fig. 1d).

**Table 1:** Summary of individuals per species successfully sequenced, the number of unique nucleotide alleles recovered per MHC region, the unique translated amino acids per region, and calculated metrics of average alleles per individual and nucelotide diversity. Asterisks denote metrics calculated from samples that were sequenced in Martin et al. (2022).

MHC region	Unique nucleotide alleles	Unique translated amino acids	Species	Individuals sampled per species	Average individual allele count (std. dev.)	nucleotide diversity (var.)
MHCI	162 (22 from this study)	141	Caretta caretta*	*88	*3.9 ± 1.5	*0.233 ± 0.01341
			Chelonia mydas*	*268	*3.5 ± 1.4	*0.201 ± 0.00959
			*Dermochelys coraicea*	62	2.1 ± 0.94	0.018 ± 0.00014
			*Lepidochelys kempii*	161	4.9 ± 1.2	0.225 ± 0.01274
			**Total individuals**	579		
MHCII chromosome 1	14	6	*Caretta caretta*	93	1.4 ± 0.48	0.007 ± 0.00005
			*Chelonia mydas*	141	1.5 ± 0.50	0.011 ± 0.00004
			*Dermochelys coraicea*	59	1.0 ± 0.13	0.030 ± 0.00100
			*Lepidochelys kempii*	36	1.1 ± 0.23	0.011 ± 0.00008
			**Total individuals**	329		
MHCII chromosome 14	294	286	*Caretta caretta*	91	6.3 ± 1.3	0.126 ± 0.00393
			*Chelonia mydas*	244	6.0 ± 1.7	0.160 ± 0.00610
			*Dermochelys coraicea*	62	3.4 ± 0.71	0.034 ± 0.00036
			*Lepidochelys kempii*	40	5.7 ± 1.9	0.143 ± 0.00531
			**Total individuals**	437		

Asterisks denote metrics calculated from samples that were sequenced in Martin et al. (2022).

The three *Cheloniidae* species had similar total allele counts, nucleotide diversity, and alleles per individual (Fig. 1). In contrast, *D. coriacea* had markedly lower MHCI and MHCII diversity metrics with the exception of nucleotide diversity at MHCII-01 (Fig. 1). This calculation was based on the pairwise difference between just two alleles: one unique to *D. coriacea* that was found in all *D. coriacea* individuals sequenced (Deco001_MHCIIB) and a shared allele (Caca001_MHCIIB) present in all four species. The shared allele was found in only a single *D. coriacea* individual (Table S5) and is likely inflating the measured nucleotide diversity for the MHCII-01 region. Since Caca001_MHCIIB was recovered in only one *D. coriacea* individual, we cannot rule out that its presence in *D. coriacea* is from contamination. Further sampling is needed to confirm if this allele is indeed shared across the two sea turtle superfamilies.

### Positive Selection Varies Across Chromosome 1 and Chromosome 14 MHC Regions

**Fig. 2.**
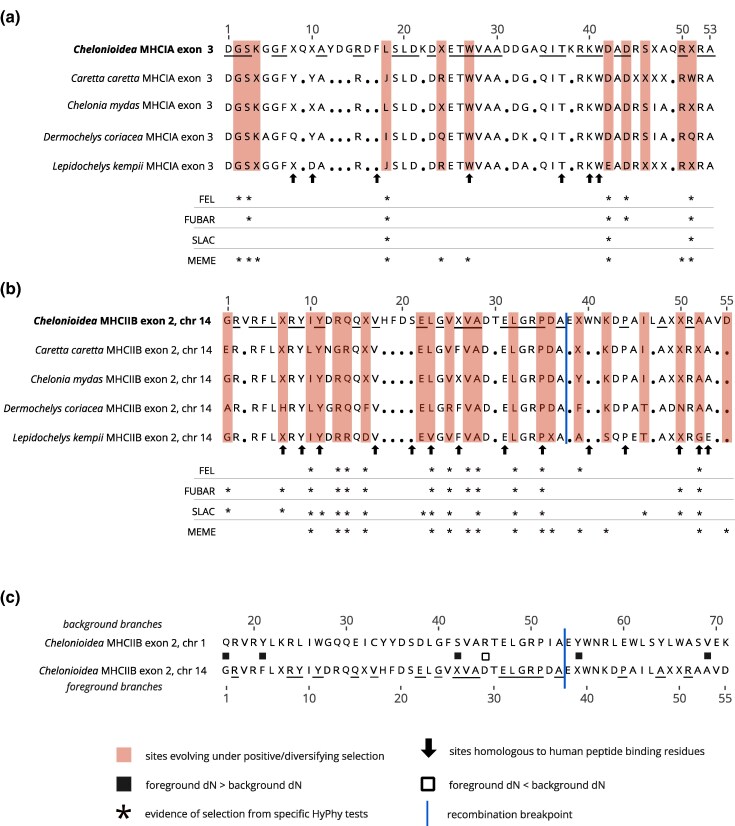
Amino acid sequence alignments of (a) MHCI exon 3 (α2 subunit) and (b) MHCIIB exon 2 on chromosome 14 (β1 subunit) alleles in four species of *Chelonioidea* (sea turtles). For each of the four species, the 50% consensus amino acid sequence is shown across all amino acid alleles originating from that species, as well as the 50% consensus from all alleles recovered in this study. Dots denote amino acid residues that were conserved across all samples. Underlined amino acid residues are those that were the most common across the species at the 50% consensus cut-off, but for which there is still amino acid variation across all alleles recovered (see Figs. S6 to S8). Arrows denote human peptide binding residues inferred from Bjorkman et al. (1987) and Sidney et al. (2008) (MHCI) and Brown et al. (1993) (MHCIIB). Amino acids under selection are shaded red and the MHC IIβ exon 2 breakpoint at residue 38 is marked with a blue line. Asterisks denote the tests identifying pervasive positive/diversifying selection at each site (FEL, FUBAR, SLAC) or episodic positive/diversifying selection at each site (MEME). c) Amino acid alignment of consensus sequences of MHCII IIβ exon 2 chr 1 and IIβ exon 2 chr 14. Squares denote sites identified by contrast-FEL to have different dN/dS ratios between the chromosome 1 and chromosome 14 branches, with filled-in squares representing sites where the non-synonymous substitution rate is higher on chromosome 14 branches than chromosome 1 branches, and open squares representing sites where non-synonymous substitution rate is lower at chromosome 14 branches than chromosome 1 branches.

There was no evidence of recombination breakpoints in MHCI or MHCII-01, but we detected a recombination breakpoint at the 108/109 base pair position in MHCII-14. We accounted for the recombination breakpoint in our standalone selection tests of MHCII-14 and our selection tests of the combined dataset of MHCII-14 and MHCII-01. We found 11 out of 53 (20.8%) codons evolved under positive and/or diversifying selection in MHCI, one of which is homologous to a human codon inferred to be part of the human peptide binding residues (PBRs) based on crystallographic studies (Fig. 2a). In MHCII-14, 21 out of 55 (38.1%) codons evolved under positive and/or diversifying selection, six of which were homologous with human PBRs (Fig. 2b). For MHCII-01, MEME found some evidence for episodic positive/diversifying selection (one codon, position 38). No other tests (FEL, FUBAR, SLAC) detected pervasive positive/diversifying selection at MHCII-01.

Selection analyses on the combined MHCII-01 and MHCII-14 dataset indicated that MHCII-14 has experienced intensification of positive selection compared to MHCII-01. RELAX detected an intensification (*K* > 1; partition 1: *P* = 0.000, *K* = 2.67, LR = 18.32; partition 2: *P* = 0.000, *K* = 3.75, LR = 56.36), and BUSTED found episodic diversifying selection at MHCII-14 (partition 1: *P* = 3.674e–7; partition 2: *P* = 3.708e–11). Contrast FEL detected nine codons for which the ratio of non-synonymous substitution rate to synonymous substitution rate (dN/dS) was different across background (MHCII-01) versus foreground (MHCII-14) branches. For eight of these codons, the rate was higher in the foreground compared to the background branches (Fig. 2c).

### Different Patterns of MHC Evolution Among Species and Loci

**Fig. 3.**
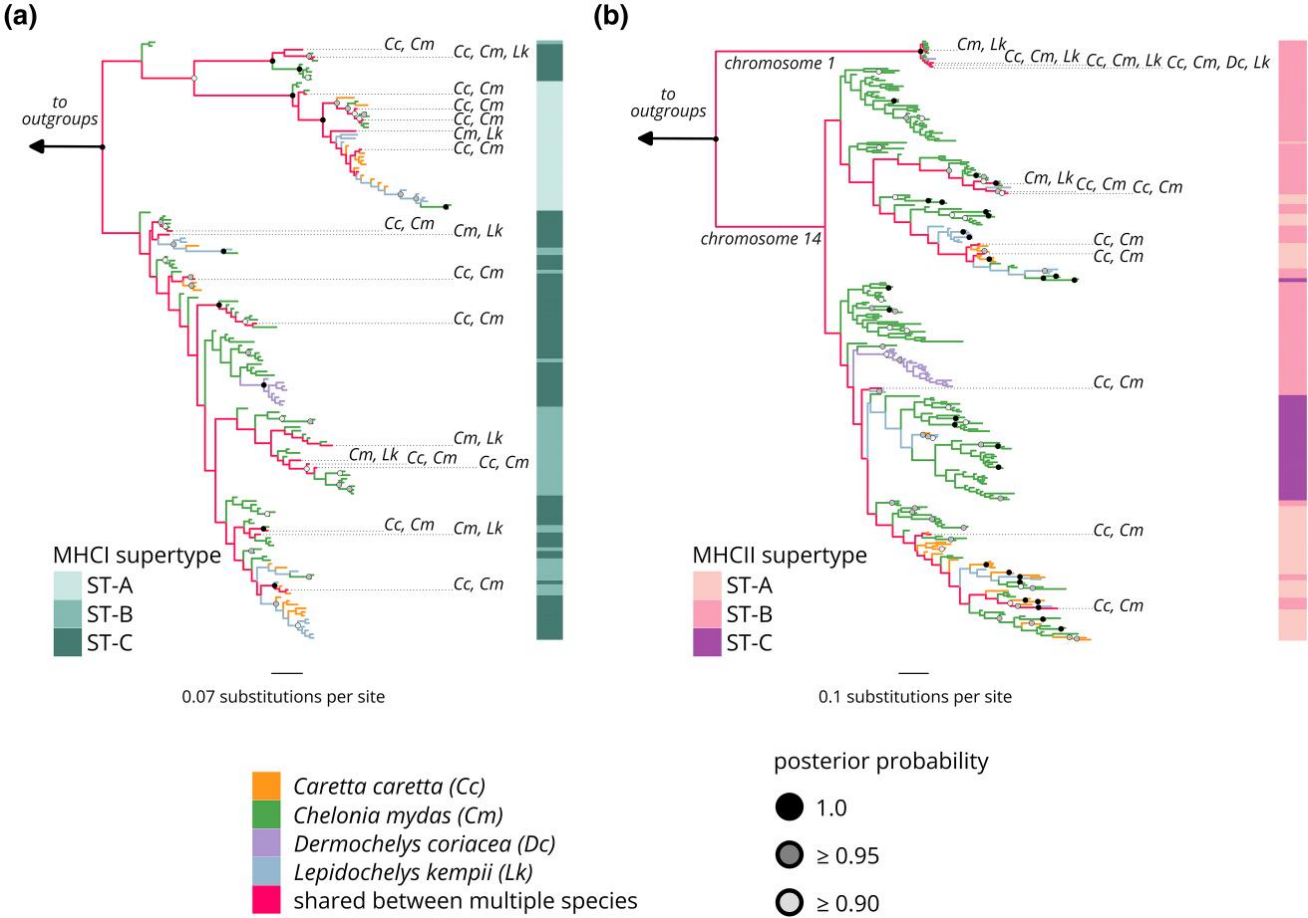
Bayesian gene trees of a) major histocompatibility complex Iα exon 3 alleles and b) IIβ exon 2 alleles recovered across four species of sea turtles. Node color denotes posterior probability support. Colored bars denote supertype membership for each allele. Branches are colored according to the species in which the allele was recovered. For alleles shared between species, the species they are found in are denoted with species abbreviations. The branch coloration for multi-species alleles is based on ancestral state, as inferred by the package ggtree.

For both MHCI and MHCII, the topology of the maximum likelihood gene tree was consistent with that of the Bayesian gene tree (Fig. S5a and b). Alleles in our Bayesian gene trees did not form monophyletic groups by species in the three *Cheloniidae* species, and numerous alleles were shared among these species (Fig. 3). While we cannot assign alleles to specific MHC gene copies due to our degenerate primers and the high polymorphism of the sequenced exons, we can suggest the minimum number of gene copies based on the number of alleles recovered per individual (Fig. 1d). A maximum of seven MHCI alleles were recovered per individual for the *Cheloniidae* species, indicating at least four MHCI gene copies, whereas *D. coriacea* in our study only had a maximum of four MHCI alleles per individual, indicating at least two gene copies. MHCI alleles grouped into two well-supported clades in the Bayesian gene tree (posterior probability >0.90) (Fig. 3a), although there were no deep well-supported clades in the Maximum Likelihood gene tree (bootstrap >90%; Fig. S5a). Each clade contains multiple paralogs from *Ca. caretta*, *Ch. mydas*, and *L. kempii* (Fig. 3a), but *D. coriacea* alleles were only recovered in one of these allele clades. More alleles were shared between *Ch. mydas* and the other two *Cheloniidae* species than between *Ca. caretta* and *L. kempii* (Fig. 1f).

For MHCII-14, the *Cheloniidae* species had a maximum of nine to 11 alleles per individual, suggesting at least five to six MHCII-14 gene copies. In *D. coriacea*, a maximum allele count of five MHCII-14 alleles indicates at least three MHCII-14 gene copies. MHCII-14 alleles formed a single well-supported clade separate from the MHCII-01 alleles in both the Bayesian gene tree (posterior probability >0.90; Fig. 3b) and Maximum Likelihood gene tree (bootstrap >90%; Fig. S5b), with paralogs from all species except *D. coriacea* present in all subclades. For MHCII-01, a maximum of two alleles were recovered per individual for all four species, suggesting a single gene copy. MHCII-01 alleles formed a well-supported basal clade in the Bayesian and Maximum Likelihood MHCII gene tree and were shared among all four species including *D. coriacea* (Fig. 3b; Fig. S5b). The only species to have any MHCII-01 alleles that were unique to their species were *Ch. mydas* and *D. coriacea* (Fig. 1f). MHCI and MHCII alleles were each functionally categorized into three supertypes, and alleles did not form monophyletic clades by supertype in our gene trees except for ST-A in the MHCI tree, which is present in just one clade (Fig. 3a). MHCII-01 was categorized into only one supertype (ST-B), a functional category that it shared with some MHCII-14 alleles. In both gene trees, supertypes were shared across all species, indicating that similar functional MHC categories exist in all species. *Dermochelys coriacea* MHCI and MHCII-14 alleles each formed a well-supported monophyletic clade (Fig. 2). These MHCI and MHCII-14 alleles each belonged to a single supertype (ST-C and ST-B, respectively), but these supertypes were also found in the other species.

### Co-ancestry Scenario Supported in MHCI and MHCII-14

**Fig. 4.**
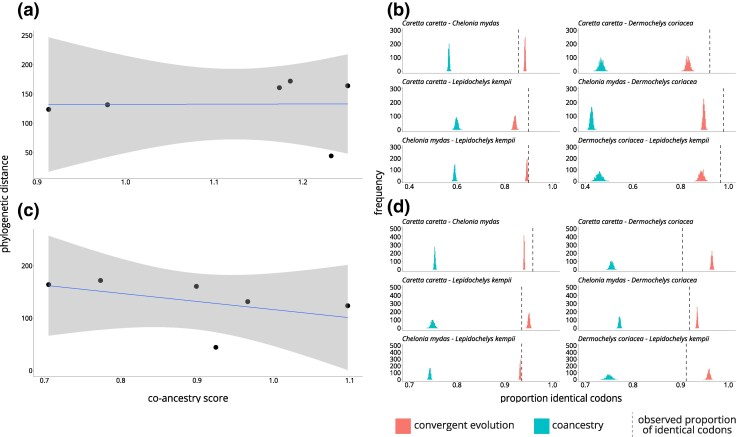
The relative contribution of co-ancestry versus convergent evolution to allelic similarity between four sea turtle species at two MHC regions. Regression of co-ancestry score and phylogenetic distance for all pairwise comparisons of species for a) MHC Iα exon 3 and c) MHC IIβ exon 2 chromosome 14. Gray area indicates linear smoothing of the regression line. Expected codon similarity distributions under convergent (blue) and co-ancestry (pink) scenarios with observed codon similarity (dotted line) for each species pairwise comparison for b) MHC Iα exon 3 and d) MHC IIβ exon 2 chromosome 14.

At both MHCI and MHCII-14, the observed proportions of identical codons shared between species at positively selected sites were higher than expected under the convergent evolution scenario for all pairwise comparisons (one tailed z-tests, false discovery rate corrected *P* < 0.05, Fig. 4b). In some cases, the observed proportion was also significantly below the co-ancestry scenario, but in all cases, the observed proportion was qualitatively closer to the co-ancestry distribution than the convergent evolution distribution. There was no relationship between co-ancestry score and phylogenetic distance for MHCI (Pearson product-moment correlation coefficient; *r* = 0.009, *P* = 0.9866; Fig. 4a). We found a slightly negative relationship between co-ancestry scores and phylogenetic distance for MHCII-14 (Fig. 4c), as expected under a co-ancestry scenario, but this was not significant (Pearson product–moment correlation coefficient; *r* = −0.457, *P* = 0.3632).

## Discussion

Here, we provide the first study on MHCII allelic variation in four of the world's seven extant sea turtle species and the first comparison of MHC immune gene regions in any turtle species. Using chromosome-specific amplicon sequence data from over 300 individuals, we find that MHC alleles shared across sea turtle species are best explained by a co-ancestry scenario, where ancestral MHC polymorphism has been maintained through speciation via balancing selection. High MHC polymorphism is likely adaptive for sea turtles given that they are long-lived and use multiple habitats throughout their life cycle, exposing them to a diverse array of pathogens. The exceptions to this pattern are *D. coriacea* and the chromosome 1 MHCII gene copy. While the three *Cheloniidae* species had high and comparable levels of MHC diversity, *D. coriacea* had low nucleotide and allelic diversity at MHCI and MHCII-14. *Dermochelys coriacea* MHC evolution was also distinct, with alleles forming species-specific clades. The atypical MHCII gene copy likewise does not evolve in an MHC-typical way; it has almost no genetic variability, does not evolve under positive selection, and has a well-supported and basal position in the gene tree, suggesting an old and functionally distinct gene. These results demonstrate that while sea turtle MHC typically evolves under balancing selection akin to many other vertebrate MHC loci (Jaratlerdsiri et al. 2012; Oliver and Piertney 2012; Minias et al. 2016; Savage et al. 2019; Talarico et al. 2021), important exceptions exist that carry conservation implications and significance for our understanding of reptile immune systems.

### Different Patterns of Diversity and Selection Among MHC Regions

We recovered strong signals of positive selection in MHCI and MHCII-14 loci, which is typical for MHC genes across vertebrates (Spurgin and Richardson 2010; Radwan et al. 2020) and has previously been documented in MHCI from *Ca. caretta* and *Ch. mydas* (Stiebens et al. 2013; Martin et al. 2022; Adkins et al. 2025). We found only marginal overlap between sea turtle amino acid sites evolving under positive/diversifying selection and human peptide binding residues (PBRs). However, many of the sea turtle sites under selection were adjacent to human PBRs, suggesting these are still key areas of the sea turtle MHC protein involved in peptide binding.

In contrast, tests of selection on MHCII-01 found only weak evidence for episodic diversifying selection at one codon position; no evidence for pervasive diversifying selection; and evidence that MHCII-01 has experienced relaxed selection relative to MHCII-14. Taken together with the low allelic diversity, basal position in the MHCII phylogeny, and the identification of a single functional supertype, the chromosome 1 copy appears to be an ancient and evolutionarily conserved MHC region in sea turtles. Furthermore, the high-quality genome assemblies of *Ch. mydas* and *D. coriacea* (Bentley et al. 2023) and the amplicon data in this study indicate that MHCII-01 is a single locus. The low allelic diversity we recover at MHCII-01 is therefore unlikely to represent primer bias, especially given that our primer pair resides in conserved intronic regions across sea turtle species (Table S1). We speculate that the role of MHCII-01 may be to present antigens to a subset of invariant T cells (Robert and Edholm 2014) or it otherwise may be a non-classical MHC locus with innate-like function (Rodgers and Cook 2005).

Of note, the presence of MHC gene copies on a different chromosome from the core MHC genomic region has been identified in the genomes of two other reptiles, the tuatara (*Sphenodon punctatus*) (Miller et al. 2015) and *Anolis* lizards (Card et al. 2022), and the functionality of the unlinked gene in these species likewise remains questionable. Future comparative MHC studies and more annotated reptile genomes will clarify whether this is a widespread phenomenon across non-avian reptiles. Unlinked MHC gene copies are likely under fewer functional constraints and may be free to evolve in divergent ways that lead to expanded or otherwise altered immune function (Siddle et al. 2009). However, few studies have characterized the evolutionary history of non-linked MHC genes in vertebrates (but, see Siddle et al. 2011; Miller et al. 2015). Future studies of non-avian reptile MHC at both regions are needed to confirm whether patterns of high allelic and nucleotide diversity only in the core MHC region are a widespread phenomenon in squamates, turtles, and crocodilians.

### MHC Diversity and Copy Number Across Sea Turtle Species

Across all three MHC regions, we find lower levels of MHC allelic and nucleotide diversity in *D. coriacea* relative to the three *Cheloniidae* species, especially at MHCI and MHCII-14. The maximum allele counts recovered in this study suggest at least two MHCI and three MHCII-14 gene copies in *D. coriacea*, compared to at least four MHCI and five to six MHCII-14 gene copies in the *Cheloniidae* species (Fig. 1d). These numbers are generally lower than the copy numbers inferred in the *Ch. mydas* and *D. coriacea* genome assemblies, which estimate 13 MHCI and 5 MHCIIB gene copies in *Ch. mydas*, and 11 MHCI and 6 MHCIIB gene copies in *D. coriacea* (Bentley et al. 2023 supplementary data). The inconsistencies between inferred copy number from maximum allele count in this study and from the genomes may arise from a few different scenarios: (i) our primers may have failed to amplify all gene copies; (ii) there was allelic dropout during amplification; or (iii) the primers amplified all copies, but the alleles were shared across the duplicated gene copies, resulting in underestimated copy counts based just on allele number. The lower nucleotide diversity and allele count per individual recovered from *D. coriacea* in this study may be an artifact of any of these above factors. The MHC is a difficult genomic region to sequence, assemble, and annotate due to its hypervariability, which may also affect copy number estimation from genomes (Vekemans et al. 2021). Future work that resolves MHC genomic architecture in sea turtles will be necessary to definitively assess copy number variation in these species.

Assessing immune gene and genomic variation in *D. coriacea* is essential to conservation efforts. The *D. coriacea* genome has lower overall genomic diversity compared to that of *Ch. mydas* and other reptiles, likely as a result of a long-term low effective population size and historic population bottlenecks (Bentley et al. 2023). In species with low genomic diversity as a result of population bottlenecks, MHC diversity is often still maintained (Aguilar et al. 2004; Oliver and Piertney 2012). In captivity, *D. coriacea* are unusually difficult to rear and often fail to thrive, with frequent mortalities from fungal and bacterial infections (Jones et al. 2000). Wild *D. coriacea* also face a number of conservation threats, including intrinsically low reproductive success (Bell et al. 2004), and mortality due to fisheries bycatch (Fossette et al. 2014, Santidrián Tomillo et al. 2017). Given the conservation threats they face, future work should quantify MHC diversity at other *D. coriacea* distinct population segments to fully assess standing immunogenetic variation in this imperiled species.

### Common Ancestry Explains Shared MHC Alleles Between Species

Despite being approximately 90 million years diverged (Phillips et al. 2022), we recovered similar and identical alleles across all four species from two families of sea turtles. The codon usage analysis shows that codons at positively selected sites that are shared between species are usually identical at the amino acid level, indicating that this ancestral polymorphism has been maintained since before speciation. At MHCI and MHCII-14, we also have evidence of pervasive positive selection and high allele number, both of which are consistent with balancing selection, which maintains alleles across species boundaries. Taken together, our results support that these shared alleles arose due to a co-ancestry scenario and not due to convergent evolution, and that they have been maintained in the descendant species due to balancing selection. Similarly to the results found by Kaesler et al. (2017), co-ancestry score was weakly negatively correlated with phylogenetic distance, suggesting that MHC allelic similarity has lessened in sea turtles as the species diverge.

However, our codon usage analyses are limited by the fact that we cannot assign alleles to specific MHCI or MHCIIB gene copies. In the future, using gene copy-specific primers spanning longer intronic segments to amplify MHC alleles will enable performing these codon usage analyses on a gene-by-gene basis within each MHC class. This approach will likely require a long-read sequencing platform for larger amplicons, but this will be important to help clarify if co-ancestry is a universal driver of similarity at all MHC gene copies in sea turtles. Furthermore, the codon usage analyses are not able to distinguish between scenarios of shared alleles from co-ancestry and those from introgression. Adaptive introgression provides an alternative explanation for shared MHC alleles between species, wherein a favorable MHC variant in one species is maintained in another species after hybridization due to novel allele advantage or pathogen-mediated selection (Hedrick 2013). Adaptive introgression of MHC variants has been demonstrated in some taxa (eg Gaczorek et al. 2023) but in practice is difficult to distinguish from distant co-ancestry without the sampling of known hybrid zones (Těšický and Vinkler 2015). Future studies of sea turtle MHC should delve more deeply into this question, especially given the prevalence of hybridization in sea turtles (Vilaça et al. 2021). The ability of sea turtles to hybridize is potentially the result of fewer physical reproductive barriers in the open ocean for these circumglobal species (Faria et al. 2021) and a very slow molecular rate of evolution that might support interspecies gametic and genomic compatibility (Avise et al. 1992). Based on whole genome resequencing data, hybridization between *Cheloniidae* sea turtle species has been occurring for millennia (Vilaça et al. 2021). Thus, a plausible explanation is that identical MHC alleles exist between species because of adaptive introgression.

We recovered identical alleles shared across *Ca. caretta*, *Ch. mydas*, and *L. kempii* at MHCI and MHCII-14. Because *Ca. caretta* and *L. kempii* are more closely related to one another than either are to *Ch. mydas* (Fig. 1a), we were surprised that fewer identical alleles were shared between *Ca. caretta* and *L. kempii* than the number shared between either species and *Ch. mydas* (Fig. 1f). This pattern might be explained by adaptive introgression, although it is not explicitly known whether *Ca. caretta* x *Ch. mydas* or *Ch. mydas* x *L. kempii* hybrids are more common than *Ca. caretta* x *L. kempii* hybrids. The uneven sharing of identical alleles may also be a result of our sampling effort. The *Ch. mydas* and *Ca. caretta* sequenced in this study are from a mixed stock juvenile foraging aggregation. While the sampling is still broadly representative of their respective north and northwest Atlantic regional management units (RMUs), our samples likely have contributions from multiple subpopulations within the north Atlantic (Reece et al. 2006; Stahelin et al. 2022). In contrast, *L. kempii* sampling was conducted in its sole RMU. More concentrated sampling of *L. kempii* MHC as well as further sampling of other RMUs of *Ca. caretta* and *Ch. mydas* (Fig. S1b and d) in future studies may reveal more shared alleles between *L. kempii* and *Ca. caretta*. Finally, we note that the identities of our genotyped alleles are based on just the peptide binding exons, so while these exons are identical, the entire gene could be different, substantially changing the degree of allele sharing in sea turtles.

## Conclusions

Our study is among the few to compare evolution of MHC regions in non-avian reptiles using a multi-species approach. We find that sea turtles possess functional diversity at classical MHC class I and II loci that has been maintained through millions of years of evolution by the forces of balancing selection. The two exceptions to this pattern are *D. coriacea* and the atypical MHCII gene copy on chromosome 1. *Dermochelys coriacea* have lower allelic and nucleotide diversity at all MHC regions compared to the other species. While *D. coriacea* is not known to be heavily impacted by the herpesvirus-associated tumor disease fibropapillomatosis, their low MHC immune gene variation suggests they may be particularly vulnerable to infectious pathogens. The atypical MHCII gene copy on chromosome 1 likely has a function separate from that of MHCII-14, given that it has low allelic and nucleotide diversity, and is evolving under relaxed selection. Future studies should confirm the function, if any, of this atypical MHC. Even in a group with low species diversity such as sea turtles, we recover different patterns of MHC diversity between MHC regions and species, suggesting that studies of additional turtle MHC may recover similarly diverse patterns. We urge more studies on non-avian reptile MHC to fill the knowledge gap of MHC evolution in ectothermic amniotes so we can understand the universality of patterns shaping vertebrate immune gene evolution.

## Materials and Methods

### Field Methods

Samples included in this study were blood, skin, or kidney tissues from *Ca. caretta, Ch. mydas*, *D. coriacea*, and *L. kempii* collected by the University of Central Florida Marine Turtle Research Group (UCFMTRG), and blood from *L. kempii* that were admitted to the New England Aquarium (NEAQ) Rescue and Rehabilitation department. All samples were taken using established field, necropsy, or rehabilitation protocols (Supplementary Methods). Until DNA extraction, skin samples in ethanol were stored at room temperature; whole blood in heparinized tubes and red blood cells without any storage buffers were stored at −20 °C; and kidney biopsies in empty cryovials were stored at −80 °C.

The *Ca. caretta* and *Ch. mydas* individuals in this study are genetic mixed stocks from the Northwest Atlantic and North Atlantic RMU, respectively (Fig. S1a–d) (Conant et al. 2009; Seminoff et al. 2015; Stahelin et al. 2022; Stahelin 2023; Wallace et al. 2023). *D. coriacea* individuals represent the Florida subpopulation of the Northwest Atlantic RMU (Fig. S1e and f) (Dutton et al. 2013; Wallace et al. 2013, 2023). All *L. kempii* samples in this study are from the sole RMU that exists for that species (Fig. S1g and h).

### DNA Extraction

We extracted DNA from skin, blood, and kidney samples according to manufacturer's instructions using either Qiagen DNeasy blood and tissue kits (Qiagen, Valencia, CA) or the New England Biolabs Monarch Genomic DNA Purification Kit (New England Biolabs, Ipswich, MA). Details are provided in Supplementary Methods.

### Primer Design

We used existing primers from Stiebens et al. (2013) to amplify MHC class IA exon 3 (hereafter MHCI) in all four sea turtle species. Previous studies (Stiebens et al. 2013; Martin et al. 2022) incorrectly referred to this as exon 2, but genetic analysis indicates clear homology to exon 3 in other reptile species. Exon 3 encodes the α2 subunit of the MHCI gene α chain, which is one of the two MHCI α subunits involved in peptide binding.

Annotations of the *Ch. mydas* (RefSeq GCF_015237465.2) and *D. coriacea* (RefSq GCF_009764565.3) genomes (Bentley et al. 2023) identify multiple putative MHC class IIB genes on chromosome 14 (five in *Ch. mydas* and six in *D. coriacea*) and a single MHC class IIB gene copy on chromosome 1. We chose to focus on MHCIIB exon 2 because this encodes for the β1 subunit of the MHCII β chain, which is involved in peptide binding. We identified all MHC class IIB exon 2 sequences from these genomes via BLAST searches using reptile MHC IIB exon 2 sequences as queries. We then aligned the sea turtle genomic MHC IIB exon 2 sequences, including >200-bp of flanking intronic sequence on 5′ and 3′ ends, and designed two sets of degenerate intron-based primers to amplify MHC class IIB exon 2 on chromosomes 1 and 14 (hereafter MHCII-01 and MHCII-14) (Table S1). The chromosome-specific nature of the primers was enabled by the fact that in the multi-species MHC alignments the flanking sequences on each respective chromosome were similar to each other and were not homologous to intronic sequences from the other chromosome.

The primers target fragments of the following sizes: 162-bp (MHCI), 289-bp (MHCII-01), and 167-bp (MHCII-14). With Illumina i7 and i5 indexing primers plus the exon-specific primers, the following fragment sizes were amplified: 377-bp (MHCI), 494-bp (MHCII-01), and 373-bp (MHCII-14) (Table S1). Our MHC primers are not specific to particular MHC gene copies so we cannot assign alleles to gene copies.

### Library Preparation

To amplify MHCI and MHCII, we used an in-house protocol based on Illumina's 16S metabarcoding protocol that utilizes two conventional polymerase chain reaction (PCR) steps. In PCR1 (ie amplicon PCR), we amplified target DNA with amplicon primers that include Illumina overhang sequences which are compatible with Illumina i7 and i5 barcoding indices. The primers for each locus are listed in Table S1, and the PCR and thermocycling conditions are described in the Supplementary Methods. In PCR2, we annealed Illumina Nextera XT sequencing adapters and i7 and i5 indices to the PCR1 product.

For each primer pair, we classified libraries semi-quantitatively, based on PCR2 band intensity (MHCI: four pools of strong, medium-strong, medium, and weak; MHCII-01 and MHCII-14: three pools of strong, medium, and weak), and pooled all PCR2 libraries based on these groups at approximately equimolar concentrations. Each pool was cleaned using a 1X solution of Sera-Mag SpeedBeads (Thermo Fisher Scientific, Waltham, MA), which was made as described in Rohland and Reich (2012). We confirmed pool quality and absence of primer dimer using an Agilent TapeStation Fragment Analyzer and then quantified each pool using a KAPA Illumina/universal library quantification kit (Roche Sequencing Systems, Pleasanton, CA) following the manufacturer's instructions. Based on the concentrations of each individual pool, we created the final library sequencing pool with equimolar concentrations of each of the gel-based groupings of libraries. We again confirmed quality using an Agilent TapeStation Fragment Analyzer and quantified it using a KAPA Illumina/universal library quantification kit (Roche Sequencing Systems, Pleasanton, CA) following manufacturer's instructions.

### Sequencing

We sequenced final pools for each locus on the MiSeq at the University of Central Florida (UCF) Genomics and Bioinformatics Core facility, with the exception of MHCI amplified from the NEAQ *L. kempii*, which were sequenced at the University of Massachusetts (UMass) Institute for Applied Life Sciences Core facility. For all runs (described in Supplementary Methods), we used 2 × 250-bp chemistry and a V2 Nano 500-cycle kit (MS-103-1003; Illumina, San Diego, CA). Starting concentrations of each library were approximately 4 nM, and we spiked in a 15% concentration of PhiX library, which is recommended for low diversity libraries like MHC.

Sequencing data for MHCI in this study were generated in three previous runs (Martin et al. 2022, BioProject PRJNA1223963), a single MiSeq run at UCF, and a single run at the UMass Institute for Applied Life Sciences Core facility. To account for run bias, we separately amplified and sequenced fifteen samples that were previously sequenced in at least one of the runs described in Martin et al. (2022). The data for MHCII-01 and MHCII-14 were generated across four and one MiSeq runs performed at the UCF Genomics and Bioinformatics Core facility, respectively. For MHCII-01, 16 individuals were separately amplified and sequenced across the runs to account for run bias, and for MHCII-14, 10 individuals were amplified in duplicate and sequenced on the same run to assess within-run variation. For MHCII-01 and MHCII-14, we recovered the same alleles in all validation libraries. For MHCI, we recovered the same alleles across runs from 3 out of 15 validation libraries. In 6 of the 12 libraries for which there was imperfect matching, libraries where we recovered fewer alleles had lower sequencing depth, which likely explains the recovery of fewer alleles in these libraries (detailed in Tables S2–S5).

### Sequence Filtering and Allele Calling

Sequencing reads were demultiplexed and trimmed of i7 and i5 indexing primers directly on the MiSeq instrument. We also trimmed off the PCR1 forward and reverse amplicon primers from paired-end raw reads using filterAndTrim() in dada2 v. 1.28 (Callahan et al. 2016) in RStudio (RStudio Team 2020). We merged reads using PEAR v. 0.9.11 (Zhang et al. 2014) using default parameters and a Phred trim quality score of 33. We used AmpliSAS (Sebastian et al. 2016) to cluster reads into MHC alleles, using the developer's recommendations for Illumina data, which includes 1% substitution error rate, 0.001% indel error rate, and minimum dominant frequency of 25%. For each primer pair, the minimum amplicon depth (number of reads per amplicon) was 100, and the minimum amplicon frequency was 10% for clustering and 3% for filtering. We compared maximum allele limits ranging from one to 14 alleles for all primer pairs. We removed any putative allele that was not 162-bp, 289-bp, and 167-bp for MHCI, MHCII-01, and MHCII-14, respectively. While sequence length polymorphism in MHC alleles can arise from indels, we did not find any evidence of it during preliminary analyses. As a result, we opted for a conservative approach to allele calling by only including alleles of these specific fragment lengths. We kept only alleles that were recovered in at least two individuals, or if they were only present in one individual, were recovered from two separate amplification and sequencing efforts. We also removed alleles that contained stop codons, or those that did not have significant BLAST hits to MHC exons. The resulting MHCII-01 alleles contained a 20-bp portion of the intron, so we removed the intronic portion of the amplified sequence and used the trimmed 269-bp exon for all downstream analyses.

### Rarefaction Analyses and Allelic Diversity

Analyses were done in R version 4.3.1 (R Core Team 2023). To assess the extent to which our results could be impacted by uneven sampling among species, we performed rarefaction analyses in the R package vegan v. 2.6-4 (Oksanen et al. 2007) using the function rarefy(). We measured nucleotide diversity using pegas v. 1.2 (Paradis 2010) and ape v. 5.7.1 (Paradis and Schliep 2019) in R for each locus separately, and plotted nucleotide diversity, alleles per individual, total alleles recovered, and total alleles recovered per total number of turtles sampled using ggplot2 v. 3.4.2 (Wickham 2016) and ggpubr v. 0.6.0 (Kassambara 2018). For MHCI nucleotide diversity, alleles per individual, total alleles recovered, and total alleles recovered per total number of turtles sampled, we only included alleles that were present in *Ca. caretta* and *Ch. mydas* sampled in Florida so that our allelic diversity results reflected only a single RMU.

### Gene Tree Reconstruction

We ran two separate alignments and phylogenetic analyses for MHCI and MHCII. We combined the MHCII-01 and MHCII-14 alleles into a single alignment and phylogenetic analysis because of their homology. For MHC class I, we included sea turtle MHC class Iα exon 3 alleles from three previous studies (Stiebens et al. 2013; Martin et al. 2022; Adkins et al. 2025). For all MHC regions, we generated the alignment in Geneious Prime v. 2023.1.2 (https://www.geneious.com) using Clustal Omega with default parameters. We trimmed each alignment to its correct open reading frame (MHCI: 159-bp; MHCII-01: 267-bp; MHCII-14: 165-bp). We then ran jmodeltest2 v. 2.1.10 (Darriba et al. 2012) on the CIPRES server (Miller et al. 2010) to determine the model of evolution that best fit the data. When the best model of evolution was not available in MrBayes (eg TPM, TIM, TVM), we used one with the most similar rate matrix that was compatible with MrBayes (eg general time reversible [GTR]). As such, the GTR model with a gamma distribution and invariant sites was used for MHCI and MHCII.

We reconstructed all gene trees using MrBayes v. 3.2.6 (Ronquist et al. 2012) on the CIPRES server. We used smooth newt (*Lissotriton vulgaris*; GenBank accession MN514000.1) and axolotl (*Ambystoma mexicanum*; GenBank accession AF156658.1) as outgroups for MHCI, and Japanese wrinkled frog (*Glandirana rugosa;* GenBank accession OQ473796.1) and High Himalaya frog *(Nanorana parkeri;* GenBank accession KR535940.1) as outgroups for MHCII. For MHCI, we ran MrBayes with two runs of four chains each for 2 × 10^7^ generations with 5 × 10^6^ discarded as burnin, sampling every 250 generations. For MHCII, we ran MrBayes with two runs of four chains each for 8 × 10^7^ generations, discarding 2 × 10^7^ as burnin, sampling every 250 generations. To confirm convergence of the MCMC chains and sufficient sampling of the posterior distribution (effective sample sizes >200 for each parameter), we used Tracer v 1.7 (Rambaut et al. 2018). We also reconstructed maximum likelihood gene trees for MHCI and MHCII using IQTree (Young and Gillung 2020) on the CIPRES web server with 1,000 ultrafast bootstraps with the -bnni option to reduce the risk of branch support overestimation in the presence of model violations, 1,000 replicates of SH-aLRT branch tests, and specified the same outgroups as above. We graphed the consensus gene trees in RStudio using the package ggtree (Yu et al. 2017).

### Selection Analyses

We ran a series of branch and site-based tests on the HyPhy server to determine if MHCI, MHCII-01, and MHCII-14 have experienced positive selection. For all individual MHC regions and the combined MHCII dataset, we ran the Genetic Algorithm for Recombination Detection (GARD) (Kosakovsky Pond et al. 2006) to identify potential recombination breakpoints, which can inflate the false positive rate of the selection tests. While GARD will provide a partitioned alignment if breakpoints are detected, it provides a neighbor-joining tree for the guide tree. We opted to generate our own partitioned alignments to reconstruct Bayesian guide trees because Bayesian methods are more accurate than neighbor-joining methods, particularly for high-diversity sequences (Holder and Lewis 2003). Thus, if breakpoints were detected, we generated partitioned alignments, assessed models of evolution, and reconstructed gene trees, as described above and analyzed each partition separately.

For each MHC region individually, we ran the following site-based tests of selection: Fixed Effects Likelihood (FEL) (Kosakovsky Pond and Frost 2005), Fast, Unconstrained Bayesian AppRoximation (FUBAR) (Murrell et al. 2013), Single-Likelihood Ancestor Counting (SLAC) (Kosakovsky Pond and Frost 2005), and Mixed Effects Model of Evolution (MEME) (Murrell et al. 2012). FEL, FUBAR, and SLAC are measures of pervasive positive/diversifying selection across all branches of the gene tree, whereas MEME is a measure of episodic positive/diversifying selection that might occur across a proportion of branches. These tests are based on the ratio of nonsynonymous substitution rate to synonymous substitution rate, or dN/dS, across branches and codon positions. After selection analyses on individual MHC regions, any codon identified as evolving under positive or diversifying selection was considered a positively selected site (hereafter PSS), and used in the codon usage analysis described below. We inferred significance at alpha less than or equal to 0.05 or at posterior probability greater than or equal to 0.95, as appropriate for each method. Since only MEME detected a single codon under positive selection in MHCII-01, we did not include position 38 in our PSS dataset for supertyping.

We also ran several tests on the combined dataset of MHCII-01 and MHCII-14 to determine if the two regions experienced different selective pressures. We ran RELAX (Wertheim et al. 2015) to see if selection was intensified or relaxed on the branches leading to MHCII-01 and MHCII-14. Because RELAX only measures whether selection is intensified and not whether it is positive/diversifying or purifying selection, we ran contrast FEL, the adaptive Branch-Site Random Effects Likelihood (aBSREL) (Smith et al. 2015), which is a branch-based test, and the Branch-Site Unrestricted Statistical Test for Episodic Diversification (BUSTED) (Murrell et al. 2015), which is a branch-site test. We ran these three tests on the combined datasets, with the MHCII-14 branches selected as test or foreground branches, to determine if there was evidence of positive or diversifying selection acting on MHCII-14 in comparison to MHCII-01.

We identified overlap between positively selected codons in sea turtles and peptide binding residues known from humans by aligning our sequences to human HLA sequences (MHCI: GenBank accession 4NQX_K; MHCII: GenBank accession AZU89091.1) Human peptide binding residues were inferred from Bjorkman et al. (1987) and Sidney et al. (2008) (MHCI) and Brown et al. (1993) (MHCIIB).

### Codon Usage Analyses

To assess whether identical alleles shared between species arise from a co-ancestry scenario or a convergent evolution scenario, we compared patterns of codon use at PSS, as inferred from the HyPhy selection analyses, in our MHCI and MHCII-14 datasets separately. We used the method implemented by Lenz et al. (2013) to generate distributions of the proportion of identical codons under the convergent evolution scenario and the co-ancestry scenario. These distributions were generated using Monte Carlo (MC) sampling and custom Perl scripts available upon request from Lenz et al. (2013).

If MHC allelic similarity between species at PSS is driven by convergent evolution, we expect that for those sites, identical amino acids shared between species will be encoded for by different codons more often than not. As such, the expected proportion of identical codons at PSS in the convergent evolution scenario will follow the overall codon frequency distribution (Lundberg and McDevitt 1992). In a pairwise fashion between species, we determined the number of identical amino acid residues and the number of identical codons across the whole exon to create a drawing pool for the MC sampling. We drew from this pool 1,000 times to generate the distribution of the proportion of identical codons expected to be shared between species under the convergent evolution scenario (CEd).

We used a similar approach to approximate the co-ancestry scenario, under which we expect that identical amino acids shared between species at PSS will be encoded by the same codon. Thus, the co-ancestry distribution of the proportion of identical codons at PSS approximates the within-species expected codon similarity across the entire exon. We again performed pairwise comparisons, but this time of allelic codon similarity within a single species, to generate a drawing pool for MC sampling. We drew from this pool 1,000 times to generate the distribution of the expected proportion of identical codons under the co-ancestry scenario (CAd). Finally, we calculated the observed proportion of identical codons at PSS between species.

We used a one-tailed *Z*-test to compare the observed proportion of identical codons between species to the mean of the distribution of expected proportions under the convergent evolution and co-ancestry scenarios. Per each MHC region, we used false discovery rate to correct *P*-values for the multiple pairwise species comparisons. To measure the relative contribution of convergent evolution and co-ancestry to identical codons shared between species, we calculated a co-ancestry score after Kaesler et al. (2017) as: (proportion of identical codons between each species—median of CEd)/(median of CAd—median CEd). We then calculated phylogenetic distances between the four species from a phylogenetic tree reconstructed from the mitochondrial control region (Phillips et al. 2022), and correlated the phylogenetic distance with the co-ancestry score for each species pair using the Pearson correlation coefficient with the function cor.test() in the stats package in RStudio.

### Supertyping

To assess functional similarity across MHC alleles, we performed supertyping using discriminant analysis of principal components on the MHCI dataset and on the combined MHCII-01 and MHCII-14 dataset. For each dataset, we generated a matrix of physiochemical descriptor variables for the amino acids at the PSS based on Sandberg et al. (1998) and then applied a two-step validation and optimization procedure to cross-validate the number of *k* clusters and the number of principal components to retain at those clusters (Martin et al. 2022). We used the R package adegenet v. 2.1.10 (Jombart 2008) to perform supertyping analyses (Supplementary Methods and Figs. S2 and S3).

## Supplementary Material

evag008_Supplementary_Data

## Data Availability

MHC allele data are deposited in GenBank under accession IDs PQ227852.1-PQ227878.1 (MHCI), PQ368784.1-PQ368797.1 (MHCII-01), and PQ227879.1-PQ228172.1 (MHCII-14). Sequencing read data are deposited in the NCBI Sequencing Read Archive under BioProject PRJNA1219623. Code, analysis files, and dataframes for this study may be found at: https://github.com/katherinermartin/Martin_et_al_2026_GBE
