# Supplementary material for: Major Histocompatibility Complex Immunogenetic Diversity Differs Substantially Across Sea Turtle Species and Genomic Regions
Source: Genome Biol Evol. 2026 Jan 23;18(2):evag008. doi: 10.1093/gbe/evag008 (PMC12891914; doi:10.1093/gbe/evag008)
Supplement: evag008_Supplementary_Data [file evag008_supplementary_data.zip › supplemental_methods_manuscript_MHC_species_loci_transfer_revised_December_clean.docx]

**Supplemental methods**

*Field sampling*

We sampled blood and skin from juvenile *Ca. caretta* and *Ch. mydas* encountered during 1995-2019 at the UCFMTRG’s central Florida USA Indian River Lagoon, Trident Turning Basin, and Indian River County sabellariid worm rock nearshore reef field sites, as described in Martin et al. (2022). These individuals represent genetic mixed stocks of the broader Northwest Atlantic and North Atlantic regional management unit (RMU) of each respective species (Figure S1A-D) [(Conant et al. 2009; Seminoff et al. 2015; Stahelin et al. 2022; Stahelin 2023; Wallace et al. 2023)](https://paperpile.com/c/XmannT/q7dN8+ve9n3+tTr0M+aeDpr+kzpjX). We also sampled skin from *D. coriacea* adult females nesting on the Archie Carr National Wildlife Refuge in Melbourne Beach, FL, USA from 2003-2022, which represent the Florida subpopulation of the Northwest Atlantic RMU (Figure S1E, F) [(Dutton et al. 2013; Wallace et al. 2013; Wallace et al. 2023)](https://paperpile.com/c/XmannT/81exP+k5LZz+kzpjX). Skin and blood from dispersal-stage juvenile *L. kempii* was sampled in the Gulf of Mexico in 2013, 2016, and 2021-2022 (Phillips et al. 2025; Phillips et al. 2022).

Kidney biopsies were sampled from *L. kempii* during the twice annual necropsy sessions held by the Florida Fish and Wildlife Conservation Commission (FWC) in May and November 2018, and June 2019. Necropsies were conducted on *L. kempii* encountered in 2017-2019 that were stranded and dead, or were live strandings that either died en route to rehabilitation or were euthanized due to their condition. All necropsies were performed by trained personnel from FWC, the National Marine Fisheries Services, or other members of the Florida Sea Turtle Stranding and Salvage Network, and kidney samples were collected by permitted UCFMTRG personnel. We also collected blood from live, cold-stunned *L. kempii* admitted to the NEAQ Rescue and Rehabilitation Department in 2018 after stranding on the shore of Cape Cod.

All blood samples were taken from the dorsal cervical sinus into heparinized blood collection tubes, using either 18- or 22-gauge needles depending on the size of the turtle. For the NEAQ *L. kempii* blood samples, we centrifuged the blood to separate the plasma from the red blood cells (RBCs). Skin samples from the *Ca. caretta*, *Ch. mydas*, and dispersal-stage *L. kempii* were taken with a 4-mm disposable Miltex biopsy punch from the trailing edge of the rear flipper and skin samples from *D. coriacea* were taken with a 4-mm disposable Miltex biopsy punch from the shoulder.

For live turtles, we first scrubbed the area to be sampled with one or more isopropyl alcohol prep pads and used QuikStop coagulant powder as necessary. Kidney biopsies were collected from necropsied strandings using 3-mm disposable biopsy punches or disposable scalpels and sterilized forceps.

*DNA extraction*

For juvenile *Ca. caretta* and *Ch. mydas*, nesting *D. coriacea*, and dispersal stage *L. kempii*, we extracted DNA from skin and blood samples using Qiagen DNeasy kits (Qiagen, Valencia, CA) following manufacturer’s protocol with the following exceptions: we incubated skin in proteinase K for 24 hours with periodic vortexing before continuing with extraction, and we eluted into 50 µL of buffer AE and then into 200 µL of buffer AE to create two eluates.

For necropsied *L. kempii*, we extracted DNA from approximately one gram of kidney tissue using the Qiagen DNeasy blood and tissue kit. We followed the manufacturer’s protocol with the following deviations: we added four to six autoclaved, 2.3-mm metal ball bearings to a sample tube with one gram of kidney tissue and homogenized it in a SPEX Sample Prep MiniG 1600 for two minutes at 1500 rpm. We then let the homogenized tissue incubate in proteinase K overnight in a thermal mixer (400 rpm). To maximize yield, DNA was eluted from the spin column with two aliquots of 100 µL of buffer AE.

For NEAQ *L. kempii*, we extracted DNA from packed RBCs using the New England Biolabs Monarch® Genomic DNA Purification Kit (New England Biolabs, Ipswich, MA), following the manufacturer’s protocol for the extraction and purification of genomic DNA from nucleated red blood cells.

*PCR*

The PCR1 reaction recipe and thermocycling conditions were identical for MHCI, MHCII-01, and MHCII-14 regions. We performed amplifications in 20-µl reactions consisting of 12.64 µl of water, 4 µl of 5X Platinum II PCR buffer (Invitrogen), 0.4 µl of dNTPs (10 mM each dNTP), 0.4 µl of 10 µM forward primer, 0.4 µl of 10 µM reverse primer, 0.16 µl of Platinum II Taq Hot-Start (Invitrogen), and 2 µl of template DNA. The thermocycling conditions were an initial denaturation at 95°C for three minutes; 40 cycles of denaturation at 94°C for 30 seconds, annealing at 54°C for 30 seconds, and extension at 68°C for 30 seconds; and a final extension at 68°C for one minute. We confirmed successful amplification of our PCR1 products on a 2% agarose gel to verify expected amplicon size.

In PCR2, (i.e., indexing PCR), we annealed Illumina Nextera XT sequencing adapters and i7 and i5 indices so that each library was indexed with unique i7 and i5 combinations. The PCR2 reaction recipe and thermocycling conditions were identical for MHCI and MHCII. We performed amplifications in 25-µl reactions consisting of 6.875 µl of water, 25 µl of 5X Platinum II PCR buffer (Invitrogen), 5 µl of dNTPs (10 mM per dNTP), 1 µl of 10 µM forward primer, 10 µl of 1 µM reverse primer, 0.125 µl of Platinum II Taq Hot-Start (Invitrogen), and 4 µl of PCR1 product as template. We amplified DNA using an initial denaturation at 95°C for three minutes; 15 cycles of denaturation at 94°C for 30 seconds, annealing at 54°C for 30 seconds, and extension at 68°C for 30 sec; and a final extension at 68°C for one minute. We confirmed successful amplification of our PCR2 products on a 2% agarose gel.

*Sequencing*

For the MiSeq runs performed at UCF, we followed manufacturer’s protocol (Illumina 15039740 Rev. A) for the denaturing and dilution of the final library, with the following exceptions: after denaturation with 0.2 N sodium hydroxide, we washed the library and PhiX control with 0.2 M tris hydrochloride because excess sodium hydroxide can inhibit flow cell-library hybridization and cause low cluster density; and we performed a final denaturation step by incubating the denatured and diluted library pool at 96°C for two minutes, after which it was placed on ice until it was loaded into the MiSeq reagent cartridge.

*Supertyping*

For each of our MHC regions, we classified MHC alleles into functionally distinct groups using discriminant principal component analysis (DAPC) to identify MHC supertypes based on the physicochemical properties of the amino acid residues (Sidney et al. 1996; Sidney et al. 2008). We created an alignment for each region consisting of the positively selected sites (PSS) as identified by selection analysis. We converted our full amino acid alignment into a matrix of five physicochemical descriptor variables per amino acid: z1 (lipophilicity or hydrophobicity), z2 (steric bulk), z3 (polarity), and z4 and z5 (electronic effects such as electronegativity and electrophilicity) (Sandberg et al. 1998), and then ran DAPC on the resulting matrix.

DAPC aims to characterize distinct clusters of genetic variation by first transforming the data via a principal components analysis (PCA) and then by identifying clusters of data variation via discriminant analysis (DA). DAPC requires groups to be identified or established prior to the analysis. In the case of MHC allele supertypes in sea turtles there is no prior knowledge about the number of functional clusters in which alleles belong to inform the number of groups to use in the analysis. Thus, it is necessary to explore both the optimal number of PCs to retain, and the optimal number of k groups (as assessed by BIC) that best describe the variation between allelic supertypes. To that end, we cross-validated the number of PCs to retain at k clusters 2 through 5 in a multi-step process using various functions in the adegenet R package.

First, k-means clustering was performed with the find.clusters() function at every k cluster 1 through 25 while retaining 50 principal component axes. This analysis was executed 10 times per cluster and the results were visualized to find the number of clusters at which the BIC was lowest. For all three regions, our analysis indicated no sharp decrease in BIC as a function of k that the range k 3 through 8 represented the sharpest decrease in BIC values as a function of k, after which BIC increased (figures S3A and S4A) and so this range of k was evaluated at all subsequent DAPC steps to identify which cluster solution was optimal for our data.

Next, k-means clustering was performed on each k 3 through 8 via the find.clusters() function, which outputs group membership for each allele at the given k. This was then used as an input for the first cross-validation analysis using the function xvalDapc() (Figure S2A, S3A which optimizes the number of PCs to retain on a training data set (comprised of 90% of the full data) by keeping the number of discriminant functions constant while varying the number of principal components retained. In this first cross-validation step, the analysis was carried out such that 1-50 principal components were retained. This process was repeated 100 times for each k cluster 3-11, the output of which indicated the optimal number of principal components to retain (based on which value minimized root mean square error [RMSE]). The second cross-validation procedure consisted of again using the function xvalDapc() in the same manner as described above, but the number of retained principal components at which xvalDapc was evaluated was centered on the number that resulted in the lowest RMSE from the previous step. For example, if 15 principal components resulted in the lowest RMSE for k = 3 in the first cross-validation step, then in the second cross-validation, the number of principal components retained for k = 3 was the range of 5 through 25. This cross-validation procedure was repeated 1000 times.

For the final step of the analysis, the discriminate principal component analysis was accomplished using the dapc() function. The output from the second cross-validation step indicated which number of retained principal components resulted in the lowest RMSE and this value was used in the DAPC analysis as the number of principal components to retain at that k. DAPC was run for each k 3 through 8 with its corresponding optimal retained principal components and the results visualized (figures S2B–2N and S3B-3N). Composition plots were used to visualize allele group membership at every k 3 through 8 such that any allele that belonged to more than one cluster was identified (figures S2C–2O and S3C-3O). See code deposited on GitHub.
