# Supplementary material for: Major Histocompatibility Complex Immunogenetic Diversity Differs Substantially Across Sea Turtle Species and Genomic Regions
Source: Genome Biol Evol. 2026 Jan 23;18(2):evag008. doi: 10.1093/gbe/evag008 (PMC12891914; doi:10.1093/gbe/evag008)
Supplement: evag008_Supplementary_Data [file evag008_supplementary_data.zip › supplemental_tables_figures_manuscript_MHC_species_loci_transfer_revised_December_clean.docx]

**Supplemental tables**

Supplemental Table 1: primer names and sequence for MHC regions in this study: MHC I exon 3, MHCIIB exon 2 chromosome 1, and MHCIIB exon 2 chromosome 14. These primers are for PCR1 (the amplicon PCR) of the metabarcoding process. The Illumina overhang sequences for forward and reverse, compatible with i7 and i5 Illumina barcodes, are also listed.

Supplemental Table 2: Summary of MHC I exon 3 amplicon depth, allele depth, allele counts, and specific alleles (with depth) recovered for validation samples.

Supplemental Table 3: Summary of MHCIIB exon 2 chromosome 1 amplicon depth, allele depth, allele counts, and specific alleles (with depth) recovered for validation samples.

Supplemental Table 4: Summary of MHCIIB exon 2 chromosome 14 amplicon depth, allele depth, allele counts, and specific alleles (with depth) recovered for validation samples.

Supplemental Table 5: Dataframe of raw allele counts and allele frequencies for MHC I exon 3, MHCIIB exon 2 chromosome 1, and MHCIIB exon 2 chromosome 14 recovered per species. Per each MHC exon sequenced, frequencies are calculated as the number of individuals per species where the allele is present divided by the total number of individuals from that species that were sampled.

Supplemental Table 6: Raw p-values and FDR-corrected p-values for pairwise species comparisons of codon usage under convergent evolution scenario and co-ancestry scenario. P-values were corrected per each MHC region (MHC I exon 3, MHCIIB exon 2 chromosome 14).

**Supplemental figures**

**Supplemental figure 1**: Sampled and global regional management units (RMU) of the four species in this study, as defined by Wallace et al. [(2023)](https://paperpile.com/c/XmannT/kzpjX/?noauthor=1). The RMUs sampled in this study for *Caretta caretta* (**A**), *Chelonia mydas* (**C**), *Dermochelys coriacea* (**E**) and *Lepidochelys kempii* (**G**) with specific sampling site denoted. Note that for *Ca. caretta* (**A, B**) and *Ch. mydas* (**C, D**), we included in our analyses alleles from two previous studies which sampled different regional management units than the ones shown on the maps (*Ca. caretta*: Stiebens et al. 2013, n = 23 unique alleles from Northeast Atlantic RMU; *Ch. mydas*: Adkins et al. 2025, n = 11 unique alleles from North Central Pacific RMU). The global distribution of RMUs are shown for each species (**B**, **D**, **F**, **H**), with the sampled RMU (saturated color) highlighted in context of the other RMUs (transparent color). RMU shapefiles accessed from SWOT/OBIS-SEAMAP 11 April 2024 [(Halpin et al. 2009)](https://paperpile.com/c/XmannT/CPeyq).

**Supplemental figure 2**: Supertyping of MHCI major histocompatibility complex Iα exon 3. Results of K-means at every k 1 through 25 while retaining 50 principal component axes. Note that the “elbow” of the graph, which usually denotes optimal value of BIC clusters to use in subsequent analysis, is not completely apparent and so the values of k for which BIC values decrease the most (*k* = 3 through 8) were evaluated (**A**). For each *k,* a DAPC was performed. A scatter plot of the principal components and composition plot of the probabilities of assignment of alleles to distinct clusters were visualized for *k* = 2 (**B, C**); *k* = 3 (**D, E**); *k* = 4 (**F, G**); *k* = 5 (**H, I**); *k* = 6 (**J, K**); *k* = 7 (**L, M**); *k* = 8 (**N, O**).

**Supplemental figure 3**: Supertyping of major histocompatibility complex IIβ exon 2 chromosome 1 and major histocompatibility complex IIβ exon 2 chromosome 14. Results of K-means at every k 1 through 25 while retaining 50 principal component axes. Note that the “elbow” of the graph, which usually denotes optimal value of BIC clusters to use in subsequent analysis, is not completely apparent and so the values of k for which BIC values decrease the most (*k* = 3 through 8) were evaluated (**A**). For each *k,* a DAPC was performed. A scatter plot of the principal components and composition plot of the probabilities of assignment of alleles to distinct clusters were visualized for *k* = 2 (**B, C**); *k* = 3 (**D, E**); *k* = 4 (**F, G**); *k* = 5 (**H, I**); *k* = 6 (**J, K**); *k* = 7 (**L, M**); *k* = 8 (**N, O**).

**Supplemental figure 4**: Rarefaction analysis of recovered alleles per species (color) for major histocompatibility complex I exon 3 (**A**), major histocompatibility complex IIB exon 2 chromosome 1 (**B**), and major histocompatibility complex II exon 2 chromosome 14 (**C**)


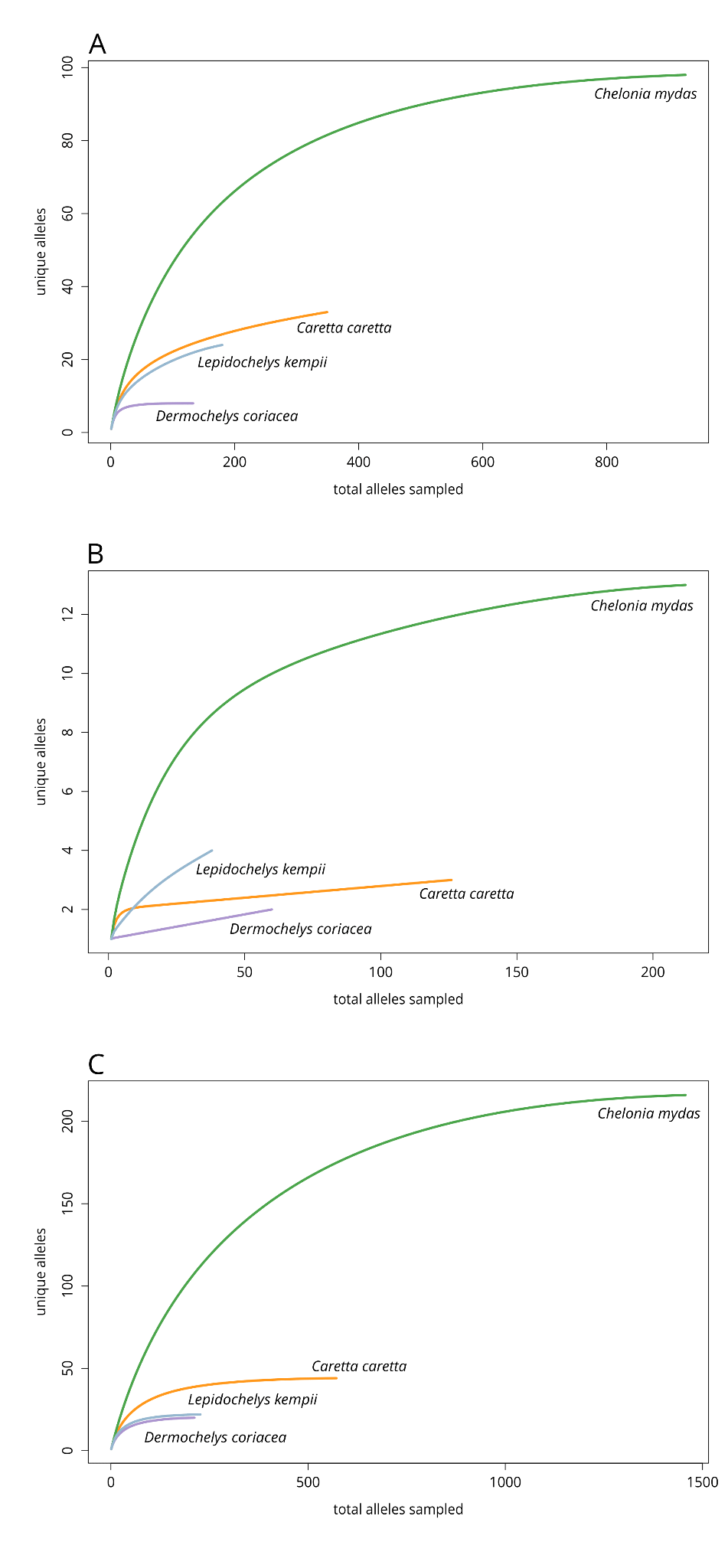


**Supplemental figure 5**: Maximum likelihood gene tree of major histocompatibility complex I exon 3 **(A)** and IIB exon 2 **(B)** alleles recovered across four species of sea turtles. Black nodes denote ultrafast bootstrap values greater than or equal to 0.90. Colored bars denote supertype membership for each allele. Branches are colored according to the species in which the allele was recovered. The branch coloration for multi-species alleles is based on ancestral state, as inferred by the package ggtree.

**Supplemental figure 6**: Amino acid sequence alignments of MHCI exon 3 (α2 subunit) in four species of *Chelonioidea* (sea turtles). The consensus sequence (top) represents bases matching at least 50% of the sequences and disagreements with the consensus are highlighted in color.

**Supplemental figure 7**: Amino acid sequence alignments of the MHCIIB exon 2 (β1 subunit) gene copy on chromosome 1 in four species of *Chelonioidea* (sea turtles). The consensus sequence (top) represents bases matching at least 50% of the sequences and disagreements with the consensus are highlighted in color.

**Supplemental figure 8**: Amino acid sequence alignments of the MHCIIB exon 2 (β1 subunit) gene copy on chromosome 14 in four species of *Chelonioidea* (sea turtles). The consensus sequence (top) represents bases matching at least 50% of the sequences and disagreements with the consensus are highlighted in color.
